# Preliminary Single-Center Experience of Bromelain-Based Eschar Removal in Children with Mixed Deep Dermal and Full Thickness Burns

**DOI:** 10.3390/jcm11164800

**Published:** 2022-08-17

**Authors:** Tomasz Korzeniowski, Ewelina Grywalska, Jerzy Strużyna, Magdalena Bugaj-Tobiasz, Agnieszka Surowiecka, Izabela Korona-Głowniak, Magdalena Staśkiewicz, Kamil Torres

**Affiliations:** 1 Chair and Department of Didactics and Medical Simulation, Medical University of Lublin, 20-093 Lublin, Poland; 2 East Center of Burns Treatment and Reconstructive Surgery, 21-010 Leczna, Poland; 3 Department of Experimental Immunology, Medical University of Lublin, 20-093 Lublin, Poland; 4 Chair and Department of Plastic, Reconstructive Surgery and Burn Treatment, Medical University of Lublin, 20-093 Lublin, Poland; 5 Department of Pharmaceutical Microbiology, Medical University of Lublin, 20-093 Lublin, Poland; 6 Center for Innovation and Accreditation, Medical University of Lublin, 20-093 Lublin, Poland

**Keywords:** wound healing, burns, enzymatic debridement

## Abstract

Introduction: Early eschar removal is the standard management of burns. The goal is to remove all of the necrotic tissue and render the wound suitable for healing or skin grafting. The enzymatic debridement of burn wounds allows for minimally invasive removal of burn eschar. The aim of the study was to describe and compare the demographic characteristics, surgical treatment and outcomes of patients treated with Nexobrid^®^ with patients who had standard surgical excision. Material and Methods: A retrospective review was conducted on children who underwent enzymatic debridement. The study group was compared with children treated with the standard of care (SoC). Results: Twelve children (mean age 8 years, range 3 to 15 years) with mixed deep dermal and full thickness burn wounds were treated with Nexobrid^®^. The mean size of the burns was 29% TBSA. The median percentage TBSA debrided using Nexobrid^®^ was 15% (range 2–27%). In a clinical assessment, enzymatic debridement was effective in removing dead tissue in a single application. No adverse reaction to Nexobrid^®^ and serious complications after enzymatic procedure were recorded in the study group. The estimated relative risk of the need for reconstructive procedures decreases 3.5 times for the study group (RR 3.5, 95%CI 0.9–13.5, *p* = 0.089). Conclusion: The bromelain-based enzymatic method offers a good and safe debridement option to improve the treatment and life quality of children with severe burns. The main outcome of interest was the number of reconstructive procedures due to scar contractures, which was reduced in the group treated enzymatically compared to the SoC-treated children.

## 1. Introduction

Early burn wound debridement is regarded as the major factor in reducing invasive burn infection and improving survival [1]. Infection is the leading cause of death in both the adults and children who survive the initial resuscitation through burn shock. Following thermal injury, the skin’s impaired barrier function, combined with the anatomical features of pediatric skin, make children more susceptible to inflammation and infection. As long as the necrotic tissue is present, the risk of infection persists. Early wound excision and coverage have become the key clinical strategy to improve burn wound care [2,3,4]. The decision to remove the eschar is critical. The most important decision-making process is therefore deciding when to operate, and at what depth to debride the wound for the optimal healing and recovery. Debridement within the first 24 h after injury significantly decreases the bacterial colonization and subsequent infection in pediatric burn patients [5,6]. The standard of care is surgical necrectomy, using sharp excision. However, the sacrifice with this method is the unintentional removal of healthy, viable tissue along with the intentional removal of the necrotic tissue. The principal drawback of the fascia excision is that it may create a considerable contour deformity. The non-excisional eschar removal methods include abrasion technique, hydro-surgery and enzymatic debridement [7]. The latter, with the use of Nexobrid^®^ rich in bromelain-based enzyme agents, is growing in popularity. The enzymatic debridement of burn wounds allows for the minimally invasive removal of necrotic tissue. Recent studies confirm the efficacy, selectivity and safety of this method [8,9]. This study aimed to describe and compare the demographic characteristics, surgical treatment and outcomes of the patients treated with Nexobrid^®^ with patients who had standard surgical excision.

## 2. Material and Methods

A retrospective chart review of the children who underwent enzymatic debridement or surgical necrectomy at the East Centre of Burns Treatment and Reconstructive Surgery in Leczna (Poland) was conducted. These were all pediatric patients with mixed deep dermal and full thickness burns treated in 2015–2020.

The enzymatic debridement was performed using Nexobrid^®^ (eschar-specific removal agent) comprising a mixture of proteolytic enzymes and bromelain (derived from the pineapple stem), according to the following protocol: thoroughly cleaning the wound with antiseptic solution and removal of dead epidermis; soaking with moisturizing dressing for 2 h; preparing and applying the Nexobrid^®^ mixture to the wound along with an occlusive dressing for 4 h; removing dissolved eschar and product remnants via scraping; wet to dry dressing with polyhexanide solution for at least 2 h.

The eschar removal procedure using Nexobrid^®^ was performed on the first day or no later than the second day after injury, under opiate or general anesthesia in the operating theatre. The vital parameters of all of the children were monitored during the anesthetic procedure and product application. 

The data collected included age, mechanism, burn characteristics, management and outcomes. The hospitalization days, additional surgical intervention and all of the complications were recorded.

The children’s characteristics were compared retrospectively with a control group, collected from the patients with the same type of injury who were treated with the standard of care (SoC). This group included the patients treated in the period before the implementation of enzymatic therapy in children in our center, or when Nexobrid^®^ was unavailable.

The double assessment of burn wounds in the study group (before and after enzymatic debridement) was used to create a two-staged treatment algorithm.

An enzymatic debridement, such as the surgical removal of necrotic tissue, carries a risk of bleeding. The patients with bleeding disorders, such as bleeding diathesis, low platelet count, established anticoagulant therapy or an increased risk of active bleeding, are at a high risk of developing complications. A critical bleeding risk evaluation was performed on each child in the study and the SoC groups.

The study received approval from the Institutional Ethics Committee of the Independent Public District Hospital in Leczna (ref. number: 01/WCLO/2021).

The statistical analysis was carried out with Tibco Statistica 13.3 (StatSoft, Palo Alto, CA, USA). The values of the parameters were presented as arithmetic means and their standard deviations (SD) or number and percentage. The normal distribution of continuous variables was tested using the Shapiro–Wilk test. The continuous variables were analyzed using the test for parametric statistics for an intergroup comparison. The distributions of the discrete variables in groups were compared with the Pearson’s chi-square test or Fisher’s exact test. The relative risk (RR) and 95% confidence interval (using the approximation of Katz) were calculated. The error was set at 5% and significance at *p*-value < 0.05.

## 3. Results

### 3.1. Descriptive Analysis of the Study Group

A total of 12 children (6 females, 6 males; mean age 9 years, range 3 to 15 years) with mixed deep dermal and full thickness burn wounds were treated with Nexobrid^®^. None of the children had any significant comorbidities and were generally in good health, except for the burns. None were excluded from the study. The mean size of the burns was 29% (range 2–64%) total body surface area (TBSA), and all (100%) were mixed burns at presentation. The majority of the burns affected the upper extremities (83%), followed by the trunk (58%) and lower limbs (42%). The burns were caused by flame (*n =* 6), scalds (*n =* 3), hot oil (*n =* 2) or contact with a hot surface (*n =* 1). The patients were treated in different settings, depending on the age, depth of the burn, extension and location of the injury. A total of 6 out of 12 children had severe burns above 25% of TBSA and were treated in the intensive care unit. Three of them required temporary mechanical ventilation. The median percentage TBSA debrided using Nexobrid^®^ was 15% (range 2–27%). In seven of the children, the enzymatic eschar removal of the entire burn surface was performed. In the remaining cases, some of the wounds qualified for conservative treatment, due to the high potential of spontaneous epithelialization in the primary assessment and the lack of a need for early surgical or enzymatic removal of necrotic tissue. The patient characteristics are summarized in Table 1.

**Figure 1:**
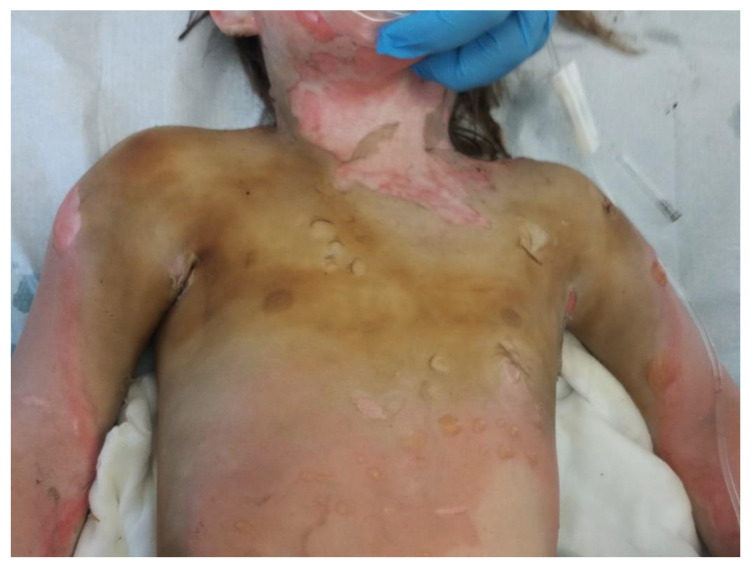
Burn on arrival.

**Figure 2:**
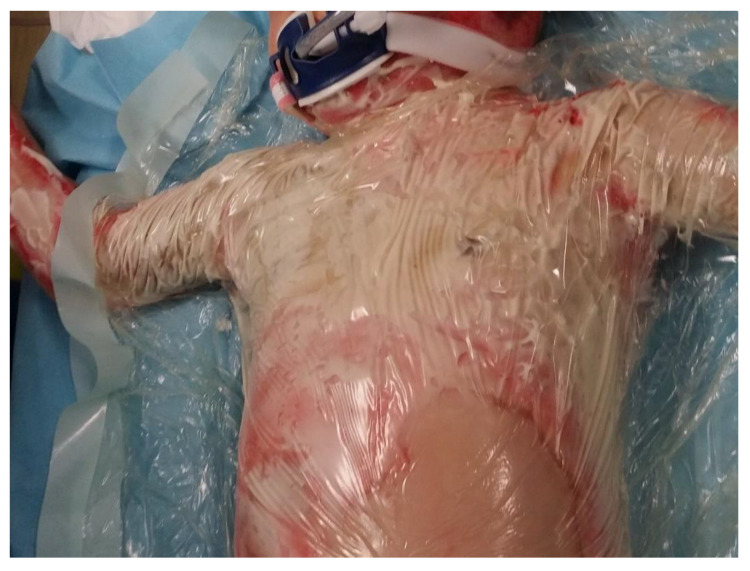
Nexobrid^®^ application.

**Figure 3:**
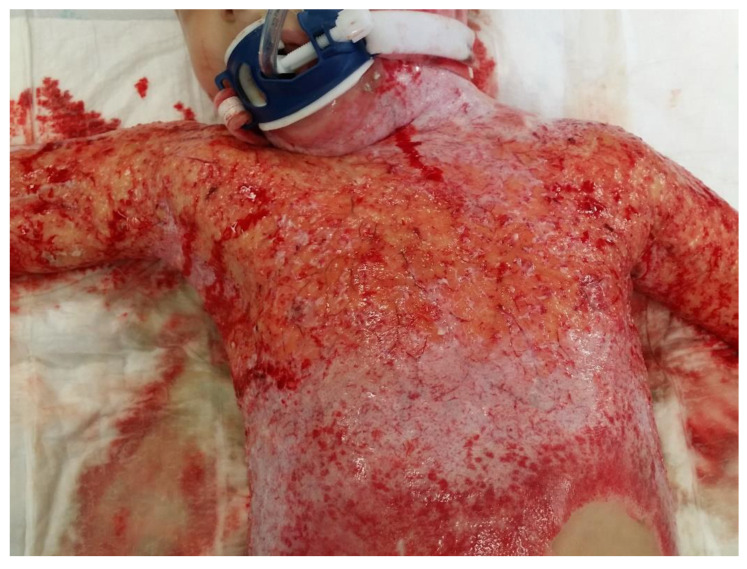
Result of enzymatic debridement.

**Figure 4:**
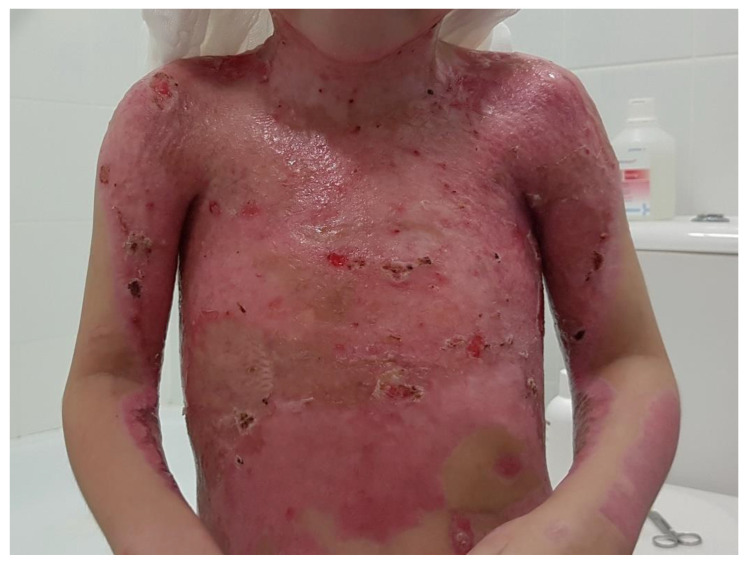
Final result (on discharge from hospital).

In a clinical assessment, the enzymatic debridement was effective in removing dead tissue in a single application in all twelve of the patients. Figure 1, Figure 2, Figure 3 and Figure 4 show the enzymatic treatment in a 5-year-old child. 

No adverse reaction to Nexobrid^®^ was recorded in the study group. Early split thickness skin grafting (STSG) was used in three of the children, due to deep burns with no chance of spontaneous healing, as assessed after enzymatic debridement. The skin grafting was performed the day after the Nexobrid^®^ application. In the other nine patients, various types of specialist dressings were used in the post enzymatic debridement wound care, to promote epithelialization. We used synthetic skin substitute (Suprathel^®^), paraffin tulle gras dressing (Jelonet^®^), wound gel with polyhexamethylene biguanide and betaine (Prontosan^®^ Gel) and silicone net dressings (Mepithel^®^), in various configurations. In the period of 14–21 days after the injury, the wound epithelialization potential was assessed. In the patients with a lack of healing progress or in the event of pseudo-eschar, additional surgical procedures were applied. A total of six of nine children required additional debridement procedures and the covering of some of the wounds with STSG.

### 3.2. Comparison to SoC group

In the SoC group, 12 children (5 females, 7 males; mean age 9 years, range 2 to 17 years) with mixed deep dermal and full thickness burn wounds were treated with standard of care (SoC). Surgical necrectomy was performed in all of the children of this group (seven hydrosurgery using Versajet^®^, four tangential excision and one fascial excision). A total of 10 of the 12 children required skin grafts (five early and five delayed). In five patients, the surgically debrided wounds were simultaneously covered with STSG. The remaining five required additional debridement procedures and skin transplantation (STSG), due to the lack of spontaneous healing progress within 14–21 days after the injury. 

The patient characteristics of SoC group are summarized in Table 2.

The study and the SoC groups were homogeneous in terms of age, type of injury, percentage of total burned area and percentage of debrided area. Wound colonization and infection rates did not differ in the pathogens identified on wound swabbing following enzymatic or surgical debridement. No significant bleeding complications were recorded in both of the groups. In the study group, hospitalization time varied from a minimum of 7 days to a maximum of 77 days, with an average of 35 days. The children in the comparison SoC group spent on average 27% more time in the hospital than the enzymatically treated patients. Although the hospitalization time of the study group is lower than that of the SoC group, the difference was not statistically significant (*p* = 0.34).

All of the children were discharged from the hospital with all of the wounds healed. In the 2-year follow-up period, two patients (17%) of the children treated with Nexobrid^®^ required secondary reconstructive procedures due to burn contractures in the hand. In one case, Z-plasty was performed, in the other, the hypertrophic scar was removed following full thickness skin grafting (FTSG). In the SoC group, as many as seven children (58%) required the release of scar contractures (three FTSG, two Z-plasty and two skin matrix Nevelia^®^). The estimated relative risk of the need for secondary reconstructive procedures increased 3.5 times for the SoC group (RR 3.5, 95%CI 0.9–13.5, *p* = 0.089).

The comparison of results between study and SoC groups is presented in Table 3.

## 4. Discussion

Early eschar removal is the standard management of deep burns. The goal is to remove all of the necrotic tissue and render the wound suitable for healing or skin grafting [10,11]. The enzymatic debridement of burn wounds is an effective way to remove eschar [12]. Subsequently covering the wound with skin substitutes, specialist dressings or skin grafts, in turn, reduces the fluid loss and metabolic requirements, and protects the wound from the external environment. Thus, early debridement and proper wound management reduce the inflammation as well as preventing the risk of infection, wound sepsis and multiple organ failure [13,14].

The current European Medicines Agency license for enzymatic debridement using Nexobrid^®^ limited its use to adults and to a maximum of 15% TBSA. The European and Polish Consensus, as well as the Italian recommendations, take into consideration enzymatic debridement for extensive burns but as an off-label use. Although, it is recommended to carry out enzymatic debridement in several stages, many of the centers in Europe had experience treating up to 25% TBSA in one session. The pretreatment risk stratification, adequate monitoring and hemodynamic support are needed when treating patients on more than 15% TBSA [15,16,17]. The literature lacks studies reporting the use of enzymatic debridement in children, with the exception of one manuscript presenting the combined experience in the use of Nexobrid^®^ in pediatric burns throughout three clinical trials, and the study that explores the different possibilities for pain management during enzymatic debridement in pediatric and adult burn patients [18,19]. 

Since 2015, the Nexobrid^®^ was added to the set of debridement tools available to the surgeon in our burn center. We gained significant knowledge during that time, starting with minor deep burns in adults. The studies confirming the safety of enzymatic debridement, as well as our experience in using this method, allowed us to extend the use of Nexobrid^®^ on larger areas and on burn wounds in children [20,21]. One of the most important advantages of enzymatic debridement using Nexobrid^®^ is its selective action. It removes eschar, leaving behind viable tissue [22,23]. The group of pediatric patients presented in this study was characterized by mixed deep dermal and full thickness burns. In this cases, it is difficult to clearly and precisely differentiate the depth, and thus the potential for spontaneous healing, or to make a decision for surgical excision and skin transplantation. Here, the enzymatic debridement appears to be the method of choice [24]. 

**Figure 5:**
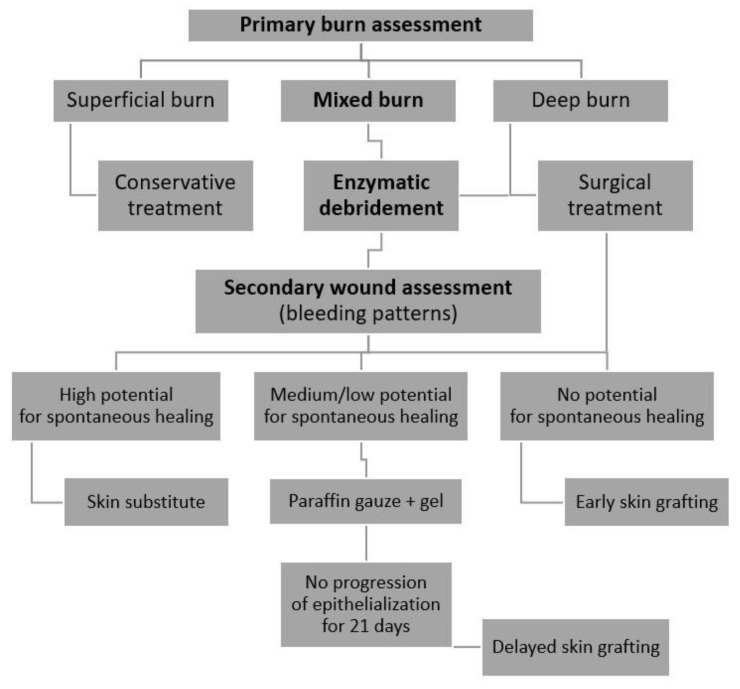
Treatment algorithm.

Enzymatic debridement is an effective diagnostic tool by assessing the wound after eschar removal, including bleeding patterns. It allows for the choice of an even better treatment strategy [25,26]. In our series, a total of only three patients underwent early autografting after Nexobrid^®^ use, compared to five patients in the control group. In most of the cases, the primary burn assessment defined wounds as deep, qualified for early surgical excision and grafting. However, after enzymatic debridement, we observed the vital layers with the dermal architecture remaining upon removal of the enzyme, which impacted on our wound management. We could apply conservative treatment, achieve spontaneous healing in some areas and reduce the need for skin transplants. Therefore, we created an algorithm adapted to our demands including a double assessment of the burn wound before and after enzymatic debridement (Figure 5).

The presented strategy may have a positive effect on the number of additional surgical procedures and the duration of treatment. The children spent an average of 44.75 days inside the hospital in the SoC group, whereas only 35.25 days in the study group. The difference, however, is not statistically significant, which is in line with the results obtained by the Italian researchers on adults. They observed a reduced amount of autologous skin grafts, when the enzymatic method was applied, but the length of stay did not show significant differences compared to the surgically treated patients [27].

The early removal of the eschar with Nexobrid^®^ is a safe and minimally invasive method. The enzymes are not harmful to healthy skin and preserve the vital dermis [21]. The study group had less skin grafts during the initial care compared to the SoC group. The number of reconstructive procedures due to scar contractures was reduced in the group treated enzymatically compared to the SoC-treated children. The children in the SoC group may have required more early skin grafting and reconstructive procedures because surgical excision is not as selective in salvaging areas of partial thickness in IIb/III burns as enzymatic debridement. Saving them from unnecessary surgery brings a significant benefit to patients and burn units. These results are in accordance with the results provided by a phase IIIb study of pediatric clinical trials. Autografting in deep partial thickness wounds was performed in 21.7% of the wounds debrided enzymatically vs. 31.8% of the wounds treated surgically (*p* = 0.44). The average long-term modified Vancouver Scar Scale scores were 3.4 for 18 wounds in 8 children treated with Nexobrid^®^ versus 4.4 for 19 wounds in 9 children debrided using surgical methods [18]. Another study on adults showed a significant difference in the scar surface appearance (modified Yeong scale), with an average score of 2.5 in the study group, in comparison with 3.2 in SoC patients [27].

Most of the research focuses on Nexobrid^®^ application to smaller areas, with particular emphasis on debriding burned hands [28,29,30,31]. Considering the safety and selectivity of this method, its use in children and severe burns seems even more justified. Recent publications addressing clinical off-label use of bromelain-based Nexobrid^®^ in more excessive burns also confirm the usefulness of this method in the intensive care setting and during burn resuscitation [32,33]. Similarly, our study did not reveal any unexpected side effects or relevant adverse events after early enzymatic debridement, irrespective of the burn area.

The research presents some limitations. The data were collected retrospectively in a single-center and it should be emphasized that the population of children was small. Our study presents a preliminary report, rather than comprehensive data, and a small study population, however we hope it provides useful clinical guidance for other burn units wishing to use enzymatic debridement in severely burned children. Future extended, comparative or randomized trials are needed to confirm our results.

## 5. Conclusion

The enzymatic debridement using Nexobrid^®^ is one of the effective methods of removing burn eschar in children, which the burn unit has at its disposal. The majority of thermal burns in children are of mixed depth, and Nexobrid^®^ has an advantage over traditional necrectomy procedures due to its selectivity in identifying the areas of partial thickness burns and preserving these areas from unnecessary skin grafting, that could improve the patient outcomes and reduce hospital costs. The two-staged treatment-algorithm presented in this study became particularly useful, in order to avoid the need for secondary surgery. The main outcome of interest was the number of reconstructive procedures due to scar contractures, which was reduced in the group that was treated enzymatically compared to the SoC-treated children.

The experience of our burn center with the Bromelain-based eschar removal in children and in high % of TBSA suggests that this method offers a good and safe option to improve the treatment and life quality of children with severe burns.

## Figures and Tables

**Table 1:** Characteristic of the study group treated enzymatically using Nexobrid^®^ (NXB).

	Gender	Age	Cause	Depth	Total%	NXB %	Location of NXB	After NXB	STSG	Reconstruction	Length of Stay
1	M	12	flame	II/III	45%	9%	upper limbs	Skin grafts	yes (early)	no	38
2	F	5	flame	II/III	27%	27%	trunk, upper limbs	Skin grafts	yes (early)	z-plasty	77
3	F	3	scald	II/III	60%	20%	lower limbs	Suprathel	no	no	82
4	M	5	hot oil	II/III	39%	12%	trunk, lower limbs	Suprathel	yes (delayed)	z-plasty	33
5	F	3	scald	II/III	20%	20%	trunk, upper limbs	Suprathel	yes (delayed)	z-plasty	25
6	M	15	flame	II/III	2%	2%	upper limb	Jelonet + Gel	no	no	7
7	F	8	hot oil	II/III	7%	7%	trunk, upper limb	Jelonet + Gel	yes (delayed)	z-plasty	24
8	M	13	flame	II/III	47%	18%	trunk, limbs	Jelonet + Gel	yes (delayed)	z-plasty	37
9	F	10	flame	II/III	64%	25%	upper and lower limbs	Jelonet + Gel	yes (delayed)	z-plasty	28
10	M	12	flame	II/III	15%	15%	trunk, upper limb	skin grafts	yes (early)	no	29
11	M	9	scald	II/III	15%	15%	trunk, limbs	Suprathel	yes (delayed)	z-plasty	35
12	F	7	contact	II/III	7%	7%	upper limb	Mepithel	no	no	8

**Table 2:** Characteristic of the SoC group treated surgically.

	Gender	Age	Cause	Depth	Total%	Necrectomy%	Location of Necrectomy	Necrectomy	STSG	Reconstruction	Length of Stay
1	M	13	flame	II/III	57%	29%	trunk, limbs	fascial	yes (early)	ftsg	104
2	M	10	contact	II/III	4%	4%	trunk, upper limb	Versajet	yes (delayed)	no	21
3	M	7	flame	II/III	13%	10%	trunk, upper limb	Versajet	yes (delayed)	ftsg	28
4	M	3	flame	II/III	20%	10%	upper limbs	Versajet	yes (early)	z-plasty	39
5	F	4	scald	II/III	42%	15%	upper, lower limbs	tangential	yes (early)	matrix	78
6	M	17	flame	II/III	39%	18%	trunk, limbs	Versajet	yes (delayed)	z-plasty	36
7	M	8	scald	II/III	23%	5%	trunk, upper limbs	Versajet	yes (delayed)	ftsg	31
8	F	5	scald	II/III	7%	4%	upper limb	Versajet	no	no	27
9	M	13	flame	II/III	15%	15%	lower limbs	tangential	no	no	25
10	F	6	flame	II/III	74%	31%	head, trunk, limbs	Versajet	yes (early)	no	47
11	M	2	contact	II/III	2%	2%	upper limb	Versajet	yes (delayed)	matrix	44
12	F	15	flame	II/III	33%	24%	trunk, upper limbs	tangential	yes (early)	no	57

**Table 3:** Comparison of results between study and SoC groups.

	Total (*n =* 24)	Study Group (*n =* 12)	SoC Group (*n =* 12)	*p*-Value
Age (mean ± SD)	8.54 ± 4.38;	8.5 ± 4.01;	8.58 ± 4.96;	0.96
Gender (*n*, %)				0.68
- male	14 (58.3)	6 (50.0)	8 (57.1)	
- female	10 (41.7)	6 (50.0)	4 (33.3)	
Hospitalization (days) (mean ± SD)	40.0 ± 23.75;	35.25 ± 22.98;	44.75 ± 24.53;	0.34
Total burned area (%) (mean ± SD)	28 ± 21;	29 ± 21;	27 ± 22;	0.86
Debrided area (%) (mean ± SD)	14 ± 9;	15 ± 8;	14 ± 10;	0.82
Cause of a burn (*n*, %)				0.36
flame	13 (54.2)	6 (50.0)	7 (58.3)	
scald	6 (25.0)	3 (25.0)	3 (25.0)	
hot oil	2 (8.3)	2 (16.7)	0 (0)	
contact	3 (12.5)	1 (8.3)	2 (16.7)	
Skin grafting (STSG)				0.79
early	8 (33.3)	3 (25.0)	5 (41.7)	
delayed	11 (45.8)	6 (50.0)	5 (41.7)	
Reconstruction (*n*, %)	9 (37.5)	2 (16.7)	7 (58.3)	0.09

## Data Availability

Not applicable.
